# Folate Receptor-Beta Has Limited Value for Fluorescent Imaging in Ovarian, Breast and Colorectal Cancer

**DOI:** 10.1371/journal.pone.0135012

**Published:** 2015-08-06

**Authors:** Esther de Boer, Lucia M. A. Crane, Marleen van Oosten, Bert van der Vegt, Tineke van der Sluis, Paulien Kooijman, Philip S. Low, Ate G. J. van der Zee, Henriette J. G. Arts, Gooitzen M. van Dam, Joost Bart

**Affiliations:** 1 Department of Surgery, Division of Surgical Oncology, University of Groningen, University Medical Center Groningen, Groningen, The Netherlands; 2 Department of Medical Microbiology, University of Groningen, University Medical Center Groningen, Groningen, The Netherlands; 3 Department of Pathology and Molecular Biology, University of Groningen, University Medical Center Groningen, Groningen, The Netherlands; 4 Department of Chemistry, Purdue University, West Lafayette, Indiana, United States of America; 5 Department of Gynaecological Oncology, University of Groningen, University Medical Center Groningen, Groningen, The Netherlands; 6 Department of Surgery, Nuclear Medicine and Molecular Imaging and Intensive Care, University of Groningen, University Medical Center Groningen, Groningen, The Netherlands; King's College London, UNITED KINGDOM

## Abstract

**Aims:**

Tumor-specific targeted imaging is rapidly evolving in cancer diagnosis. The folate receptor alpha (FR-α) has already been identified as a suitable target for cancer therapy and imaging. FR-α is present on ~40% of human cancers. FR-β is known to be expressed on several hematologic malignancies and on activated macrophages, but little is known about FR-β expression in solid tumors. Additional or simultaneous expression of FR-β could help extend the indications for folate-based drugs and imaging agents. In this study, the expression pattern of FR-β is evaluated in ovarian, breast and colorectal cancer.

**Methods:**

FR-β expression was analyzed by semi-quantitative scoring of immunohistochemical staining on tissue microarrays (TMAs) of 339 ovarian cancer patients, 418 breast cancer patients, on 20 slides of colorectal cancer samples and on 25 samples of diverticulitis.

**Results:**

FR-β expression was seen in 21% of ovarian cancer samples, 9% of breast cancer samples, and 55% of colorectal cancer samples. Expression was weak or moderate. Of the diverticulitis samples, 80% were positive for FR-β expression in macrophages. FR-β status neither correlated to known disease-related variables, nor showed association with overall survival and progression free survival in ovarian and breast cancer. In breast cancer, negative axillary status was significantly correlated to FR-β expression (p=0.022).

**Conclusions:**

FR-β expression was low or absent in the majority of ovarian, breast and colorectal tumor samples. From the present study we conclude that the low FR-β expression in ovarian and breast tumor tissue indicates limited practical use of this receptor in diagnostic imaging and therapeutic purposes. Due to weak expression, FR-β is not regarded as a suitable target in colorectal cancer.

## Introduction

The folate receptor (FR) has been proposed as a target in cancer therapy and imaging. The vitamin folate (B9) and its synthetic form folic acid, are indispensable for nucleotide synthesis. Under physiologic conditions, uptake of folate occurs mostly through the reduced folate carrier (RFC), which is sufficient in healthy tissues despite its fairly low affinity for folate [1]. Furthermore, some healthy tissues and a number of pathologic processes express the transmembrane FR which has in the past two decades attracted attention as a target for diagnostic and therapeutic compounds because of its much higher affinity for folate [2].

The FR gene family includes four isoforms: FR-α, FR-β, FR-δ and FR-γ. The latter two play a role in regulatory T-cells and fall outside the scope of this article. FR-α is expressed on the apical side of a number of epithelial cells and is present on ~40% of solid tumors. Expression varies between tumors, showing high expression in serous ovarian cancer and renal carcinoma versus low to moderate expression in breast, colorectal and lung cancer [3–5]. The value of FR-α targeting in cancer diagnosis and therapy has been shown using folate-conjugated imaging agents as well as folate-based drugs [6–8].

Little is known regarding the expression of FR-β in solid tumors. Studies using mRNA isolation and isolation of cellular membranes demonstrated expression on activated but not resting macrophages, as well as on the surfaces of malignant cells of hematopoietic origin such as acute leukemia [9,10]. Targeting of FR-β has shown to be feasible in the visualization of inflammatory processes in rheumatoid arthritis and atherosclerosis [2,8,11].

Thus far, it is largely unknown whether FR-β is also expressed on solid tumors, apart from one article stating that this may indeed be the case [6]. However, one study indicates that FR-β mRNA can be found in tumors suggesting that FR-β may play a role in tumor cell growth and metastasis. It is suggested that the mechanism of action is mainly via infiltration of tumor-associated macrophages (TAMs), which are guided towards the tumor by cytokines and are thought to induce a more malignant tumor behavior [12]. It has furthermore been suggested that folate-based immunotherapy for cancer may also exert its effect by targeting TAMs.

The overexpression of FR-α and/or FR-β on cancer cells and myelogenous cells allows for both non-invasive diagnostic imaging of FR-positive cancers and inflammatory processes, and subsequent treatment using folate-based drugs or FR-targeting antibodies. For instance, discrimination could be made between a sigmoidal malignant neoplasm and diverticulitis. However, the limited and variable expression of FR-α on solid tumors is a major restriction for FR-targeted approaches. Additional or simultaneous expression of FR-β could help extend the indications. The aim of this study is to investigate the expression pattern of FR-β in ovarian, breast and colorectal tumors, and thereby evaluate the possibilities and limitations for cancer specific imaging and therapy in these cancer types.

## Materials and Methods

### Patient tissue samples

All study specimens were collected from our own archives of the Department of Pathology and Medical Biology at the University Medical Center Groningen (UMCG). Tissue microarrays (TMAs) of ovarian cancer (339 cases) and breast cancer (418 cases) were available from earlier studies, including full databases of anonymized patient data. All TMAs were constructed in the UMCG as described before [13,14], and consisted of 4 cores per patient. Furthermore, twenty patient tumor samples of colorectal cancer were selected, all collected during cytoreductive sugery combined with hyperthermic intraperitoneal chemotherapy (HIPEC). Twenty-five patient samples of diverticulitis were selected based on postoperative pathology reports. Patient data were retrieved from the electronic hospital patient data system and from the Dutch Pathology Registry (PALGA). All patient data were anonymized and all studies concerning this databank were conducted in accordance with the rules and regulations posed by our institutional medical ethical research board; [The Medical Ethical Committee (in Dutch: Medisch Ethische Toetsingsingscommissie or METc) of the University Medical Center Groningen (UMCG)] which approved the study. Since we used archival pathology material, which does not interfere with patient care and does not involve the physical involvement of the patient, no ethical approval is required according to Dutch legislation [the Medical Research Involving Human Subjects Act (Wet medisch-wetenschappelijk onderzoek met mensen, WMO [15])], and as such informed consent was not required.

### Sample preparation and immunohistochemistry

FR-β expression was determined by immunohistochemistry (IHC). The antibody for FR-β staining (anti-human FR-β; Biotin-m909) was kindly provided by professor P.S. Low (Purdue University, West Lafayette, IN, USA). TMAs were cut into 3 μm sections and fixed on glass slides. All specimens were fixed by 10% formalin and embedded in paraffin. The samples were rinsed well in distilled water after the formalin-fixed, paraffin-embedded (FFPE) samples were deparaffinized with 3 changes of xylene and rehydrated in a series of alcohols. Antigen retrieval was achieved by placing the slices in a preheated Target Retrieval Solution (Dako, Glostrup, Denmark) for 5 minutes at 125°C then cooled in the buffer for 20 minutes to 90°C followed by a 5 minute rinse in running phosphate buffered saline (PBS). Subsequently, sections were incubated with 0.3% H_2_O_2_ in PBS for 30 minutes to inactivate the endogenous peroxidases. Slides were rinsed well and then incubated for 3 hours at room temperature with the Biotinylated- hu @ hu FR-β at a 1:100. After washing with PBS, the sections were incubated with peroxidase labeled streptavidin (Thermo Scientific, Waltham, MA, USA and Dako) for 30 minutes. To visualize peroxidase activity, the sections were incubated in 3,3-diaminobenzidine (DAB+) for 10 minutes. Subsequently, sections were counterstained with haematoxylin, mounted with a permanent mounting media and coverslipped. Placental tissue was used as a positive control, as earlier studies have described that placental villous stroma tissue should be positive [16]. In addition tissue macrophages should be positive for FR-β, so they served as internal positive control. Their presence was confirmed using the anti-CD68 monoclonal antibody PGM 1 in a subset of 5 diverticulitis samples. Since obvious negative tissues are not described the same (placental) tissue was used as a negative control. Specimen handling was identical, except application of the primary antibody.

### Histological analysis

#### Breast cancer and ovarian cancer

TMA cores were considered representative if they contained more than 25% tumor.

Patient samples were included if 2 or 3 samples were considered representative.

**Fig 1:**
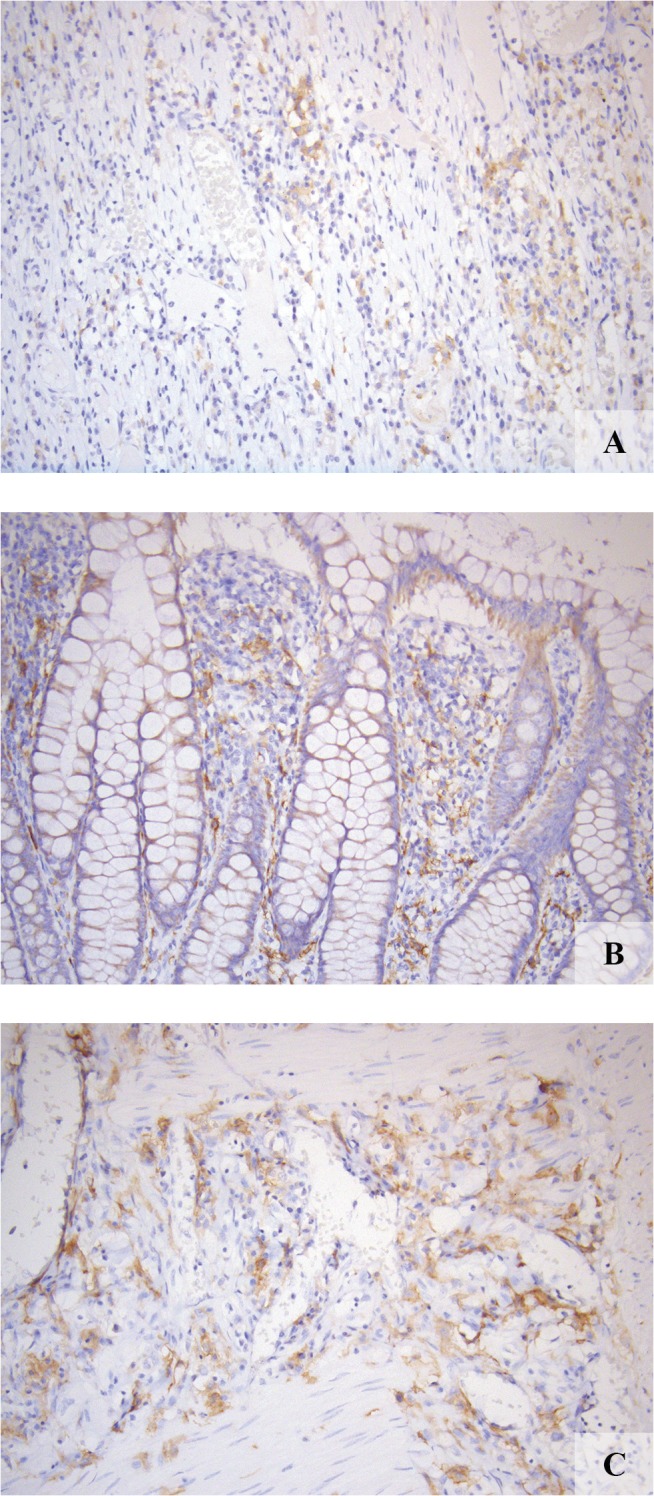
FR-β expression in representative samples. *A*: weak expression (staining intensity 1). *B*: moderate expression (staining intensity 2). *C*: strong expression (staining intensity 3).

All TMA’s and slides were graded for FR-β staining (0 = no staining; 1 = weak staining (Fig 1A); 2 = moderate staining (Fig 1B); 3 = strong staining (Fig 1C)) when at least 25% of the core consisted of tumor cells. The scoring was performed according to previously published studies [2,4]. After judging of the cores separately, a mean score was appointed to each patient sample (consisting of four cores) based on the overall staining intensity observed in the sample.

#### Colorectal cancer and diverticulitis

Colorectal cancer samples and diverticulitis samples were scored based on the dominant staining intensity. Finally, for statistical purposes, all cases were divided into a ‘negative staining’ group (score 0) and a ‘positive staining’ group (score 1, 2 or 3). Scoring was performed by the researchers (LMAC, EDB, PK) after training by an experienced pathologist (JB), and all samples were confirmed by the pathologist. Scores were compared, and in cases of different grading, discussed until consensus was met.

In this study, the colorectal cancer cases showed expression of FR-β in a subset of tissue macrophages. To elucidate whether this was a tumor-specific phenomenon, and therefore an interesting target for colorectal cancer specific optical imaging, an additional set of diverticulitis samples (*n = 25)* were stained to evaluate the presence of FR-β on inflammatory associated macrophages.

### Statistical analysis

All statistical analyses were performed by using SPSS 21.0 (SPSS Inc, Chicago, IL, USA). Descriptive statistics were calculated for variables of interest. The Chi-square test was applied to analyze correlations between FR-β staining and several disease-related variables. Survival analyses were performed for ovarian cancer and breast cancer. Due to small numbers, survival could not be calculated for colorectal cancer. Overall survival was defined as the time from diagnosis until the last follow-up while alive or death due to the tumor (ovarian or breast, respectively). Progression-free survival was defined as the time from primary surgery until progression of the disease, recurring disease or last follow-up. Survival curves were generated using Kaplan-Meier analysis. The prognostic influence of FR-β expression was tested in a Cox proportional hazards model. P-values <0.05 were considered statistically significant.

### Image capture

The images shown in this paper were acquired using a Leica DM4000B microscope and a Leica DFC450 digital camera (Leica Microsystems GmbH, Wetzlar Germany).

## Results

### Validation staining technique

**Fig 2:**
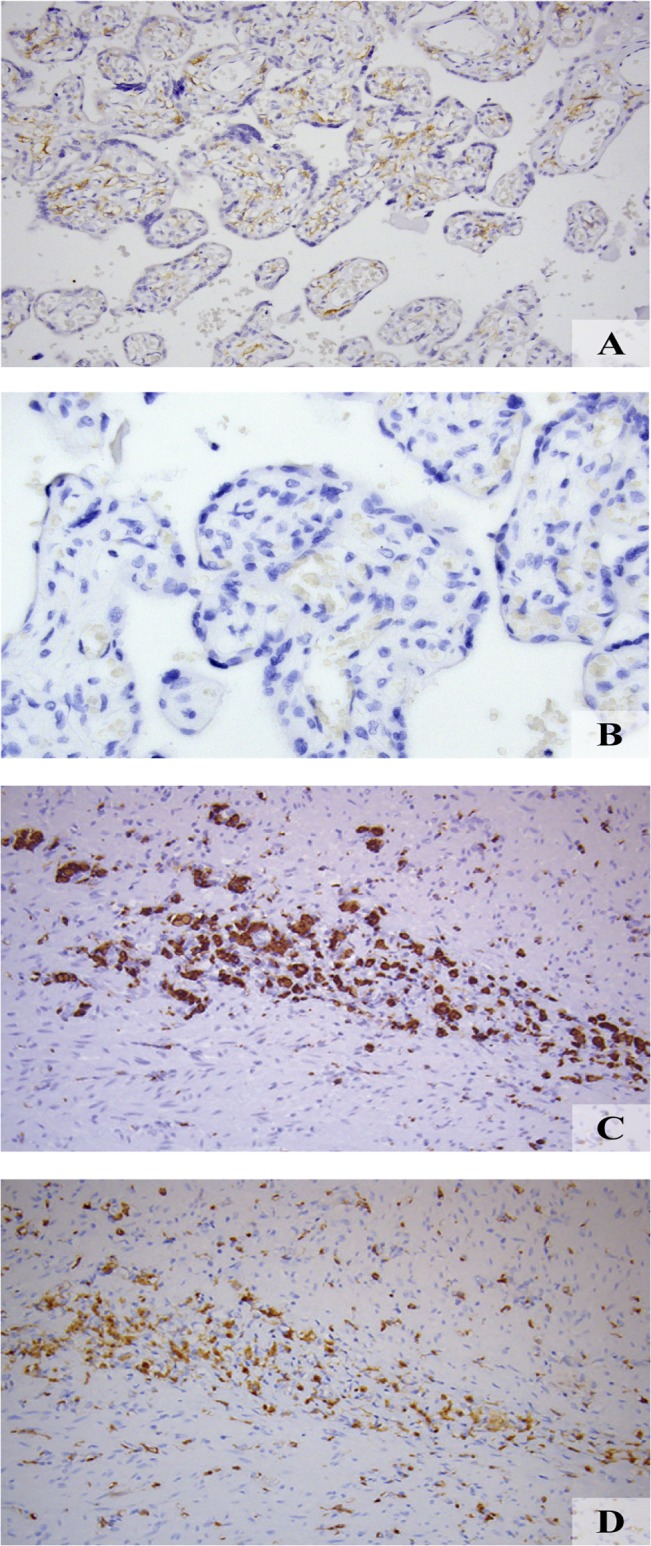
Validation anti-human FR-β specificity and immunoreactivity. *A*: Strong staining was appreciated in the placental villous stroma known to express FR-β, validating anti human FR-β binding specificity and immunoreactivity *B*: Absent staining was shown after staining with the secondary antibody only. Co-expression of the *C*: anti-CD68 stain and *D*: FR-β in activated macrophages in a diverticulitis sample validated anti-human FR-β specificity and immunoreactivity.

Strong staining was shown to be present in the placental villous stroma (Fig 2A). Staining was shown to co-localize with the so-called Hoffbauer cells, which are considered specialized macrophages, known to express FR-β**.** Staining with the secondary antibody only demonstrated absent staining (Fig 2B). Furthermore, the anti-CD68 macrophage staining (Fig 2C) was shown to accurately co-localize with the FR-β stain (Fig 2D), validating specificity and immunoreactivity of the anti-human FR-β antibody.

### Ovarian cancer

#### Patient characteristics

Baseline patient characteristics of the 339 patients included in the database are shown in Table 1 and are summarized here. Histology was known in 326 of 339 cases. The total number of tumor samples obtained from these 326 patients was 386 (primary surgery n = 295, interval debulking surgery n = 63 and surgery for recurrent disease n = 28). The majority of tumors consisted of serous carcinoma (n = 198; 58.4%), followed by endometrioid (n = 49; 14.5%) and mucinous adenocarcinoma (n = 38; 11.2%). More than seventy percent of tumors were classified as FIGO stage III or IV (n = 243; 71.7%).

**Table 1:** Characteristics ovarian cancer patients.

	N = 339		
**Age (mean, min-max, SD)**	57.4	16–89	13.2
**Age; grouped**	**n**	**%**	
<58 years old	158	46.6	
≥ 58 years old	180	53.1	
Missing	1	0.3	
**Tumor type**	**n**	**%**	
Serous adenocarcinoma	198	58.4	
Other	106	31.3	
*- mucinous adenocarcinoma*	38	11.2	
*- endometrioid adenocarcinoma*	49	14.5	
*- clear cell carcinoma*	18	5.3	
*- undifferentiated*	1	0.3	
Missing	35	10.3	
**FIGO-stage**	**n**	**%**	
Stage I	66	19.5	
Stage II	28	8.3	
Stage III	192	56.6	
Stage IV	51	15.0	
Missing	2	0.6	
**Tumor differentiation grade**	**n**	**%**	
Grade I	55	16.2	
Grade II	91	26.8	
Grade III	153	45.1	
Undifferentiated	15	4.4	
Missing	25	7.4	
**Progression**	**n**	**%**	
Yes	203	59.9	
No	132	38.9	
Missing	4	1.2	
**Progression free survival (months)**			
**(mean, min-max, SD)**	29.6	0–207	36.3
**Overal survival (months)**			
**(mean, min-max, SD)**	54.0	0–134	33.0
**Residual disease**	**n**	**%**	
< 2 cm	157	46.3	
≥ 2 cm	154	45.4	
Missing	28	8.3	

#### FR-β expression

**Fig 3:**
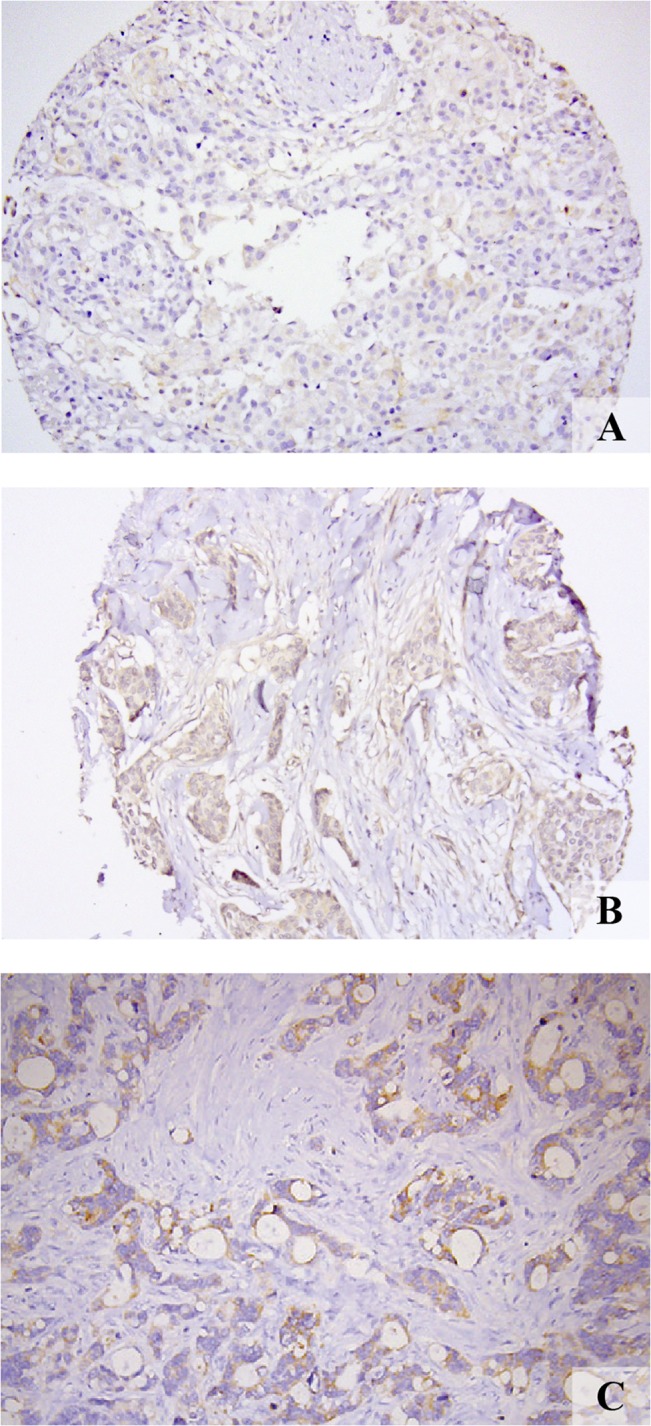
FR-β expression. Representative images of weak staining intensities in (*A)* ovarian, (*B)* breast and (C) colorectal cancer samples.

Representative FR-β staining was obtained in all 339 patient samples included in our study of which almost 80% showed no FR-β expression at all (n = 269; 79.4%), 18.6% (n = 63) showed weak staining and only 2.1% (n = 7) showed moderate staining (Fig 3A). None of the samples showed strong staining.

FR-β expression was evaluated in regard to known risk factors in ovarian cancer; e.g. grade of differentiation, FIGO stage, histological subtype (serous vs. non-serous), age (<58 vs. ≥58) and residual disease ≥2 cm. None of these factors showed a significant correlation to FR-β status.

In a previous study, FR-α expression was analyzed using the same tumor samples and database [2]. The correlation found in the present study between FR-α and FR-β expression was weak, but not significant (p = 0.098).

#### Survival analysis

All of the above factors were associated with reduced progression free survival (PFS) and overall survival (OS). In multivariate regression analysis, the only significant predictors of both PFS and OS were FIGO stage III-IV (p<0.0001), residual tumor >2 cm (p<0.0001) and serous histology (p = 0.045). FR-β expression did not show any correlation with either PFS or OS.

### Breast cancer

#### Patient characteristics

Baseline patient characteristics of all 418 patients included in the database are presented in Table 2 and will only be summarized here. All patients were female, of whom 48.1% were ≥ 59 years of age. Mean tumor size was 22.7 mm (1–140 mm). Ratios of breast-conserving surgical therapy and mastectomy were nearly equal (50.7% and 49.0%, respectively); 63.4% of patients received adjuvant radiotherapy and 49.5% received adjuvant chemotherapy. Nearly all tumors were diagnosed as either invasive ductal carcinoma (50.8%) or invasive ductal carcinoma with ductal carcinoma in situ (42.3%).

**Table 2:** Characteristics breast cancer patients.

	N = 418		
**Age (mean, min-max, SD)**	59.7	27–91	13.8
**Treatment interventions**	**n**	**%**	
Breast-conserving surgery	212	50.7	
Mastectomy	205	49.0	
Adjuvant radiotherapy	265	63.4	
Adjuvant chemotherapy	207	49.5	
**Age; grouped**	**n**	**%**	
<59 years old	217	51.9	
≥59 years old	201	48.1	
**HER2-neu status**	**n**	**%**	
Negative	254	60.8	
Positive	149	35.6	
Missing	15	3.6	
**Progesterone receptor status**	**n**	**%**	
Negative	140	33.5	
Positive	247	59.1	
Missing	31	7.4	
**Pathological tumor size**			
**(mean, min-max, SD in mm)**	22.7	1–140	18.0
**Tumor differentiation grade**	**n**	**%**	
Grade I	105	25.1	
Grade II	180	43.1	
Grade III	131	31.3	
Missing	2	0.5	
**Axillary status**	**n**	**%**	
Negative	214	51.2	
Positive	191	45.7	
missing	13	3.1	
**Time relapse free**			
**(mean, min-max, SD)**	50.6	0–134	33.0
**Overall survival(months)**			
(mean, min-max, SD)	54.0	0–134	33.0

#### FR-β expression

FR-β scores could be obtained in all 418 cases included in our study. The vast majority of tumor samples showed no FR-β staining (n = 381; 91.1%), whereas 36 samples (8.6%) showed weak staining and only 1 sample (0.002%) showed moderate staining (Fig 3B). For statistical analyses, weak and moderate scores were combined into an ‘FR-β positive’ group, as compared to the ‘FR-β negative’ group. Low rates of positive staining were observed in invasive ductal carcinoma (n = 20; 4.8%). There was no FR-β staining in other histological subtypes.

FR-β expression showed no significant correlation to age, differentiation grade, tumor size, relapse rate or HER2-neu status. Interestingly, axillary status showed a significant correlation with FR-β, with a higher expression rate in negative axillary lymph nodes compared to positive nodes (p = 0.022)(Table 2).

#### Survival analysis

In a multivariate regression analysis, differentiation grade III (p = 0.009) was the only significant predictor for overall survival. A trend was observed for positive axillary status (p = 0.080). Predictors for progression free survival were high differentiation grade (p = 0.005), positive axillary status (p = 0.024) and positive progesterone receptor status (p = 0.017). FR-β status showed no correlation to either OS or PFS.

### Colorectal cancer

#### Patient characteristics

Twenty tumor samples were stained for FR-β, all obtained during cytoreductive surgery for colorectal cancer combined with hyperthermic intraperitoneal chemotherapy (HIPEC). Of these, thirteen were female and seven were male, and the mean age was 54.6 (range 34–75). Tumor differentiation was good in six patients, moderate in six patients and poor in eight patients. There were twelve mucinous adenocarcinomas, seven intestinal type adenocarcinomas and one singlet cell carcinoma.

#### FR-β expression

Eleven of 20 (55%) tumor samples stained positive for FR-β: however, staining intensity was variable, ranging from weak to faintly visible (Fig 3C). Of the eleven positive samples, six tumors were classified as mucinous adenocarcinoma (50% of total number of this tumor subtype) and five were intestinal type adenocarcinoma (71% of total number of this tumor subtype). Most staining was seen at the apical side of the tumor cells. In addition, tissue macrophages showed expression of FR-β. In the surrounding adipose tissue, weakly positive staining was seen in one sample, while no expression was observed in fifteen samples. All blood vessels were negative. Six samples contained smooth muscle cells, of which one sample scored positive and five scored negative. Healthy intestinal epithelium stained weakly positive in five samples. Two samples contained ovarian tissue, and both were negative for FR-β. The small number of samples did not allow for survival analyses.

### Diverticulitis

#### Patient characteristics

Seventeen of 25 patients included were female (68%). The mean age of all patients was 64 (range 29–86). Most patients underwent a sigmoid resection because of diverticulosis or suspected malignancy. One patient needed surgery because of widespread endometriosis.

#### FR-β expression

Of the 25 diverticulitis samples, 20 (80%) stained positive, of which moderate or strong FR-β expression was seen in nine samples (45%), and weak expression in eleven samples (55%). FR-β staining was shown to co-localized predominantly with the macrophages. The five negative samples were diagnosed as a submucosal abcess, non-active colitis, endometriosis with a few diverticula, and, in two cases, active diverticulitis.

## Discussion

The aim of this study was to evaluate the expression of the FR-β in ovarian, breast and colorectal cancer. In summary, FR-β expression was low or absent in almost all tumor samples of ovarian, breast and colorectal cancer, with staining seen in 21%, 9% and 55%, respectively. Furthermore, FR-β status showed no association with either OS or PFS in ovarian and breast cancer.

Several studies report FR-β expression in myelogenous leukemias of hematopoietic origin [17,18]. Sega et al reported FR-β expression on a variety of solid tumors of diverse origins [6]. Conversely, our data show low or absent FR-β expression in most of the investigated ovarian, breast and colorectal tumor samples.

Previously, FR-α expression in ovarian cancer was analyzed using the same tumor tissue samples and database [2]. No significant correlation could be detected between the FR-α and FR-β expression (p = 0.098). FR-β expression was also evaluated in regard to known risk factors in ovarian and breast cancer. None of the known risk factors for ovarian cancer showed a significant correlation to FR-β status. Likewise, FR-β expression showed no significant correlation to most known risk factors for breast cancer. Axillary status was the only factor that showed significant correlation to FR-β expression. A higher FR-β expression rate was seen in the tissue microarrays (TMAs) of breast cancer patients with negative axillary lymph nodes compared to positive nodes (P = 0.022). A careful hypothesis might be that FR-β expression could be used as prognostic biomarker to indicate long-term outcome of breast cancer patients. However, the overall low expression rate of only 9% hampers further conclusions. The transmembrane folate receptor (FR) expressed by cancer cells, in particular the alpha subtype, has proven to be a useful target for non-invasive diagnostic imaging and therapy [2,7]. From the present study we conclude that the low FR-β expression in ovarian and breast tumor tissue implies little practical use of this receptor in diagnostic imaging and for therapeutic purposes.

The relationship between inflammation and cancer is widely appreciated [19]. Chronic inflammation is thought to increase the risk of cancer and activated macrophages contribute to a worse prognosis by enhancing matrix degradation and metastasis [20,21]. Our results demonstrated that colorectal cancer cases showed expression of FR-β in a subset of tissue macrophages. However, since 80% of the diverticulitis samples also demonstrated FR-β expression in a comparable amount of the macrophages, this indicates that FR-β positivity in tissue macrophages near an infiltrative tumor is not a tumor-specific phenomenon. Therefore, FR-β staining in tissue macrophages is probably not useful to discriminate between an inflammatory process and a malignant neoplasm.

## Conclusions

FR-β expression was low or absent in the majority of ovarian, breast and colorectal tumor samples. From the present study we conclude that the low FR-β expression in tumor tissue implies limited practical use of this receptor in diagnostic imaging and therapeutic purposes in ovarian, breast cancer and colorectal cancer. In addition, these findings do not support the possibility that FR-β could be used as a target to discriminate between colorectal cancer and diverticulitis.

## References

[1] Antony AC. Folate receptors. Annu Rev Nutr 1996;16: 501–521. 8839936 10.1146/annurev.nu.16.070196.002441

[2] Low PS, Henne WA, Doorneweerd DD. Discovery and development of folic-acid-based receptor targeting for imaging and therapy of cancer and inflammatory diseases. Acc Chem Res 2008;41: 120–129. 17655275 10.1021/ar7000815

[3] Parker N, Turk MJ, Westrick E, Lewis JD, Low PS, Laemon CP. Folate receptor expression in carcinomas and normal tissues determined by a quantitative radioligand binding assay. Anal Biochem 2005;338: 284–293. 15745749 10.1016/j.ab.2004.12.026

[4] Crane LM, Arts HJ, van Oosten M, Low PS, van der Zee AG, van Dam GM, et al The effect of chemotherapy on expression of folate receptor-alpha in ovarian cancer. Cell Oncol (Dordr) 2012;35: 9–18. 21647742 10.1007/s13402-011-0052-6 PMC3268989

[5] Kalli KR, Oberg AL, Keeney GL, Christianson TJ, Low PS, Knutson KL, et al Folate receptor alpha as a tumor target in epithelial ovarian cancer. Gynecol Oncol 2008;108: 619–626. 10.1016/j.ygyno.2007.11.020 18222534 PMC2707764

[6] Sega EI, Low PS. Tumor detection using folate receptor-targeted imaging agents. Cancer Metastasis Rev 2008;27: 655–664. 10.1007/s10555-008-9155-6 18523731

[7] van Dam GM, Themelis G, Crane LM, Harlaar NJ, Pleijhuis RG, Kelder W, et al Intraoperative tumor-specific fluorescence imaging in ovarian cancer by folate receptor-alpha targeting: first in-human results. Nat Med 2011;17: 1315–1319. 10.1038/nm.2472 21926976

[8] Low PS, Kularatne SA. Folate-targeted therapeutic and imaging agents for cancer. Curr Opin Chem Biol 2009;13: 256–262. 10.1016/j.cbpa.2009.03.022 19419901

[9] Ross JF, Chaudhuri PK, Ratnam M. Differential regulation of folate receptor isoforms in normal and malignant tissues in vivo and in established cell lines. Physiologic and clinical implications. Cancer 1994;73: 2432–2443. 7513252 10.1002/1097-0142(19940501)73:9<2432::aid-cncr2820730929>3.0.co;2-s

[10] Xia W, Hilgenbrink AR, Matteson EL, Lockwood MB, Cheng JX, Low PS. A functional folate receptor is induced during macrophage activation and can be used to target drugs to activated macrophages. Blood 2009;113: 438–446. 10.1182/blood-2008-04-150789 18952896

[11] Jager NA, Westra J, van Dam GM, Teteloshvili N, Tio RA, Breek JC, et al Targeted folate receptor beta fluorescence imaging as a measure of inflammation to estimate vulnerability within human atherosclerotic carotid plaque. J Nucl Med 2012;53: 1222–1229. 10.2967/jnumed.111.099671 22855837

[12] Puig-Kröger A, Sierra-Filardi E, Dominguez-Soto A, Samaniego R, Corcuera MT, Gómez-Aguado F, et al Folate receptor beta is expressed by tumor-associated macrophages and constitutes a marker for M2 anti-inflammatory/regulatory macrophages. Cancer Res 2009;69: 9395–9403. 10.1158/0008-5472.CAN-09-2050 19951991

[13] van der Vegt B, de Bock GH, Bart J, Zwartjes NG, Wesseling J. Validation of the 4B5 rabbit monoclonal antibody in determining Her2/neu status in breast cancer. Mod Pathol 2009;22: 879–886. 10.1038/modpathol.2009.37 19305385

[14] van der Vegt B, de Roos MA, Peterse JL, Patriarca C, Hilkens J, de Bock GH, et al The expression pattern of MUC1 (EMA) is related to tumour characteristics and clinical outcome of invasive ductal breast carcinoma. Histopathology 2007;51: 322–335. 17645748 10.1111/j.1365-2559.2007.02757.x

[15] Central Committee on Research involving Human Subjects (Centrale Commissie Mensgebonden Onderzoek) (text in Dutch). Available: http://www.ccmo-online.nl/main.asp?pid=10&sid=30&ssid=51.

[16] Ratnam M, Marquardt H, Duhring JL, Freisheim JH. Homologous membrane folate binding proteins in human placenta: cloning and sequence of a cDNA. Biochemistry.1989;28: 8249–54. 2605182 10.1021/bi00446a042

[17] Ross JF, Wang H, Behm FG, Mathew P, Wu M, Booth R, et al Folate receptor type beta is a neutrophilic lineage marker and is differentially expressed in myeloid leukemia. Cancer 1999;85: 348–357. 10023702 10.1002/(sici)1097-0142(19990115)85:2<348::aid-cncr12>3.0.co;2-4

[18] Shen F, Ross JF, Wang X, Ratnam M. Identification of a novel folate receptor, a truncated receptor, and receptor type beta in hematopoietic cells: cDNA cloning, expression, immunoreactivity, and tissue specificity. Biochemistry 1994;33: 1209–1215. 8110752 10.1021/bi00171a021

[19] Hanahan D, Weinberg RA. The hallmarks of cancer. Cell 2000;100: 57–70. 10647931 10.1016/s0092-8674(00)81683-9

[20] Rakoff-Nahoum S. Why cancer and inflammation? Yale J Biol Med 2006;79: 123–130. 17940622 PMC1994795

[21] Lu H, Ouyang W, Huang C. Inflammation, a key event in cancer development. Mol Cancer Res 2006;4: 221–233. 16603636 10.1158/1541-7786.MCR-05-0261

